# The Search for Hesperian Organic Matter on Mars: Pyrolysis Studies of Sediments Rich in Sulfur and Iron

**DOI:** 10.1089/ast.2017.1717

**Published:** 2018-04-01

**Authors:** James M.T. Lewis, Jens Najorka, Jonathan S. Watson, Mark A. Sephton

**Affiliations:** ^1^ Impacts and Astromaterials Research Centre, Department of Earth Science and Engineering, Imperial College London, London, UK.; ^2^ Impacts and Astromaterials Research Centre, Department of Mineralogy, Natural History Museum, London, UK.

## Abstract

Jarosite on Mars is of significant geological and astrobiological interest, as it forms in acidic aqueous conditions that are potentially habitable for acidophilic organisms. Jarosite can provide environmental context and may host organic matter. The most common extraction technique used to search for organic compounds on the surface of Mars is pyrolysis. However, thermal decomposition of jarosite releases oxygen into pyrolysis ovens, which degrades organic signals. Jarosite has a close association with the iron oxyhydroxide goethite in many depositional/diagenetic environments. Hematite can form by dehydration of goethite or directly from jarosite under certain aqueous conditions. Goethite and hematite are significantly more amenable than jarosite for pyrolysis experiments employed to search for organic matter. Analysis of the mineralogy and organic chemistry of samples from a natural acidic stream revealed a diverse response for organic compounds during pyrolysis of goethite-rich layers but a poor response for jarosite-rich or mixed jarosite-goethite samples. Goethite units that are associated with jarosite, but do not contain jarosite themselves, should be targeted for organic detection pyrolysis experiments on Mars. These findings are extremely timely, as exploration targets for Mars Science Laboratory include Vera Rubin Ridge (formerly known as “Hematite Ridge”), which may have formed from goethite precursors. Key Words: Mars—Pyrolysis—Jarosite—Goethite—Hematite—Biosignatures. Astrobiology 18, 454–464.

## 1. Introduction

There is a continuous and dedicated attempt to assess whether life could have evolved beyond Earth (Des Marais *et al.,* 2008; Grotzinger *et al.,* 2012; Phillips and Pappalardo, 2014; Beegle *et al.,* 2015; Goesmann *et al.,* 2017). Mars has been a focus for missions seeking to assess habitability and identify biosignatures, as its crust records a long and diverse history of aqueous processes (Bibring *et al.,* 2006; Des Marais *et al.,* 2008; Ehlmann *et al.,* 2008; Grotzinger *et al.,* 2012; Beegle *et al.,* 2015; Goesmann *et al.,* 2017). The surface and near-surface of Mars have transitioned through several periods dominated by the formation of different types of alteration minerals (Bibring *et al.,* 2006; Ehlmann *et al.,* 2011; Gaillard *et al.,* 2013). In the Early Noachian, wet and potentially warm conditions occurred, which led to the production of extensive phyllosilicates (Bibring *et al.,* 2006; Ehlmann *et al.,* 2011; Gaillard *et al.,* 2013). In the Late Noachian and Hesperian, acidic sulfur-rich conditions were widespread as the carbon/sulfur ratio of volcanic gases decreased substantially in response to decreasing atmospheric pressure (Bibring *et al.,* 2006; Gaillard *et al.,* 2013). The Amazonian has been mostly oxidizing, dry and dominated by nanophase iron oxides (Bibring *et al.,* 2006). Sedimentary formations hosting fine-grained clay-rich sediments or phases resistant to chemical weathering, such as silica, are known to be suited to the long-term preservation of organic matter (Farmer and Des Marais, 1999; Ehlmann *et al.,* 2008). Thus, the sampling of ancient clay-rich or silica-rich units is a high priority for organic detection missions (Farmer and Des Marais, 1999; Ehlmann *et al.,* 2008; Grotzinger *et al.,* 2014). Given the mineralogical diversity of Mars and the operational constraints of missions to the surface, it is important also to consider other martian mineral assemblages that may be promising hosts of organic matter.

Missions sent to Mars with the ability to detect organic compounds include the Viking landers, which reached the surface in 1976 (Biemann *et al.,* 1977); the Phoenix lander in 2008 (Guinn *et al.,* 2008); and Mars Science Laboratory (MSL), which landed in 2012 (Grotzinger *et al.,* 2012). The Viking gas chromatograph–mass spectrometer (GC-MS), the Phoenix Thermal and Evolved Gas Analyzer (TEGA), and MSL's Sample Analysis at Mars (SAM) instrument suite all made use of thermal extraction to analyze martian rocks and soils for the presence of organic matter (Anderson *et al.,* 1972; Biemann *et al.,* 1977; Guinn *et al.,* 2008; Grotzinger *et al.,* 2012; Mahaffy *et al.,* 2012). The Mars Organic Molecule Analyzer (MOMA) instrument on the upcoming ExoMars rover will also use thermal extraction protocols (Goesmann *et al.,* 2017). The characterization of thermal extracts usually occurs by mass spectrometry of evolved gases during ramped heating (evolved gas analysis, EGA) or through GC-MS (Biemann *et al.,* 1977; Guinn *et al.,* 2008; Mahaffy *et al.,* 2012).

The organic data obtained by pyrolysis experiments conducted on the surface of Mars are dominated by simple organochlorines (Biemann *et al.,* 1977; Glavin *et al.,* 2013; Leshin *et al.,* 2013; Ming *et al.,* 2014; Freissinet *et al.,* 2015). These compounds have an ambiguous origin and were initially considered to have most likely originated from contamination from Earth (Biemann *et al.,* 1977). Perchlorate salts (ClO_4_^-^) were first detected by the Phoenix lander in 2008 and have subsequently been found to be widespread on Mars (Hecht *et al.,* 2009; Archer *et al.,* 2013; Glavin *et al.,* 2013). Perchlorates can chlorinate and oxidize organic compounds during pyrolysis, degrading or removing their signals and complicating their detection (Ming *et al.,* 2009; Glavin *et al.,* 2013). MSL detected organochlorines at elevated levels that could not be fully explained by contamination when it analyzed mudstones in Yellowknife Bay in Gale Crater, indicating that some of the carbon in the organochlorines must be from the martian surface (Freissinet *et al.,* 2015). In addition, evidence for refractory organic matter was seen during high-temperature pyrolysis of the Yellowknife Bay and Pahrump Hills mudstones (Eigenbrode *et al.,* 2015). Organosulfur compounds, including thiophenes and thiols, have also been detected by SAM GC-MS and EGA experiments (Eigenbrode *et al.,* 2015).

Mars Science Laboratory's study site of Gale Crater hosts Aeolis Mons, a central mound where the lower stratigraphy shows a progression from clay-bearing to sulfate-bearing strata (Grotzinger *et al.,* 2012). Given the mineralogical diversity of the lower slopes of Aeolis Mons, it is important to consider whether any units outside of clay-rich or silica-rich layers are promising candidates for the detection of organic matter. The observed transition from clays to sulfates is related to the fact that sulfur degassing from lavas is influenced by pressure, oxygen fugacity, and water content (Gaillard *et al.,* 2013). As the atmospheric pressure of Mars decreased in the Late Noachian, the amount of sulfur dioxide degassed from lavas increased greatly (Gaillard *et al.,* 2013). Sulfate-forming conditions are likely to have persisted well into the Hesperian (Bibring *et al.,* 2006; Gaillard *et al.,* 2013). While deleterious to prebiotic chemistry, acidic, sulfate-rich waters are inhabited by acidophilic microbes on Earth, so it is important to consider whether similar organisms may have evolved on Mars and whether their remains could be preserved in Hesperian sediments (Squyres and Knoll, 2005).

Magnesium sulfate has been shown to trap organic matter within its mineral structure and offer protection from oxychlorine phases during pyrolysis (François *et al.,* 2016). Studies of natural samples also suggest that the iron sulfate jarosite can preserve organic compounds such as glycine (Kotler *et al.,* 2008). However, sulfates, like perchlorates, release oxygen during pyrolysis (Bailey and Smith, 1972). Sulfate ions release sulfur trioxide during thermal decomposition, which breaks down to sulfur dioxide and atomic oxygen (Bailey and Smith, 1972). The identification of organic compounds in pyrolysis data from sulfate-containing samples can therefore be problematic, particularly when analyzing samples with trace amounts of organic matter (Lewis *et al.,* 2015). Jarosite typically has a close association with the iron oxyhydroxide goethite, except in depositional or diagenetic environments with extremely low pH values or low water/rock ratios (Zolotov and Shock, 2005). Subsequent aqueous weathering of jarosite outcrops may convert the iron sulfate to goethite (Stoffregen *et al.,* 2000; Zolotov and Shock, 2005). In addition, goethite can dehydrate to hematite, and at low water activities or in solutions with high phosphate concentrations jarosite may alter directly to hematite (Zolotov and Shock, 2005; Barrón *et al.,* 2006). Though the anhydrous nanophase iron oxides that dominate present-day Mars are unlikely to be associated with organic matter, hematite and goethite formed in association with jarosite require consideration. Reactive iron phases, such as goethite, can bind to organic matter and aid its preservation (Lalonde *et al.,* 2012). Unlike sulfates, iron oxides and oxyhydroxides do not release substantial amounts of oxygen during pyrolysis (Földvári, 2011; Lewis *et al.,* 2015).

Sediments rich in sulfates and iron oxides were examined at Meridiani Planum and in Gusev Crater by the Mars Exploration Rovers (MERs) (Arvidson *et al.,* 2010, 2011). The mission aims of the MERs were to assess current and past martian environments and the role of water in the alteration of surface materials (Arvidson *et al.,* 2011). The MERs were not tasked with detecting organic compounds (Arvidson *et al.,* 2011). The aqueous oxidation of pyrite to jarosite and goethite, followed by dehydration to hematite, is one of the proposed theories for the formation of the sulfate-rich layered deposits observed at Meridiani Planum (Zolotov and Shock, 2005). Hematite concretions were present as a surface lag, and the MER-B rover Opportunity was able to section some of the spherules with its Rock Abrasion Tool (RAT), discovering that hematite was present throughout the spherules and not just on the surface (Squyres and Knoll, 2005; Zolotov and Shock, 2005; Squyres *et al.,* 2006). Measurements of a rock named Clovis from the Columbia Hills in Gusev Crater by the Spirit rover revealed significant amounts of goethite (Klingelhöfer *et al.,* 2005). X-ray diffraction (XRD) experiments by the Chemistry and Mineralogy (CheMin) instrument on MSL have detected hematite at many locations in Gale Crater (Vaniman *et al.,* 2014; Rampe *et al.,* 2016). Jarosite has also been observed at low concentrations within Gale Crater (Léveillé *et al.,* 2015). MSL's exploration targets include Vera Rubin Ridge (formerly known as “Hematite Ridge”) and hydrated sulfate units, which were identified from orbit by Mars Reconnaissance Orbiter (Fraeman *et al.,* 2013). The formation of hematite from goethite precursors is one of the potential formation mechanisms considered for Vera Rubin Ridge (Fraeman *et al.,* 2013).

**FIG. 1.**
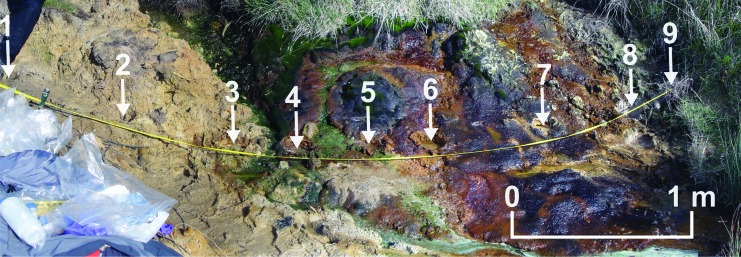
Sampling locations across the acidic stream study site. Cores are numbered 1–9. The small acidic stream can be seen passing between Core 3 and Core 4 and then flowing to the right. To the left of the stream, the soils were typically dry with a yellow tint reflecting the presence of jarosite. To the right of the stream, the soils were mostly waterlogged and generally capped with a thin goethite crust. A fibrous purple microbial mat with a distinctive surface sheen covered much of the goethite crust.

In the present study, pyrolysis and XRD experiments were conducted on natural samples collected from the sediments surrounding an acidic stream (Fig. 1). Sediments enriched in iron sulfates, iron oxyhydroxides, and iron oxides have already been studied on Mars, but never by a roving mission capable of detecting organic compounds. This may no longer be the case when MSL studies Vera Rubin Ridge. Future Mars missions are also likely to encounter iron-rich and sulfur-rich sediments. Investigating whether such mineralogies are effective hosts of organic matter and amenable to pyrolysis experiments is therefore of extreme importance.

## 2. Materials and Methods

### 2.1. Samples

Samples were collected across a 4 m study area located at the eastern end of St. Oswald's Bay, Dorset, United Kingdom (Fig. 1). Acidic waters were flowing in a small stream emanating from the pyrite-bearing Wealden group (Kemp *et al.,* 2012). The pH of the stream was measured with Ricca Chemical pH test strips from Fisher Scientific. To the west of the stream, the soils were dry and tinted yellow. To the east, the soils were waterlogged, and bright yellow jarosite layers were capped with a red goethite layer, which was itself capped by a purple microbial mat. Nine cores were taken across the study site (Fig. 1). As the alteration minerals formed near-surface layers that were only a few centimeters in thickness, the samples were extracted with a trowel to cut cores 10 cm in diameter and 10–15 cm in depth, preserving the layering and indigenous organic chemistry in the center of the core. The samples were wrapped in foil and stored in plastic bags for transport. The samples were uncovered and stored on a lab bench at room temperature until dry and then frozen.

The frozen cores were divided along their vertical axes with a solvent-cleaned saw (methanol and dichloromethane), and great care was taken to avoid disturbing the stratigraphy. The biological materials present at the top of each core were removed and analyzed separately. The mineral layers within each core were identified, separated, and then powdered in a pestle and mortar. The mineral powders were further dried in a Labconco freeze dryer for an hour.

### 2.2. X-ray diffraction

X-ray diffraction analyses were performed on a Philips PW 1830 fitted with an autosampler with the powders analyzed under copper radiation between 2.5 and 90°2θ using a step size of 0.02 with 2 s per step. Samples were back loaded onto sandpaper to reduce preferred orientation. The diffraction patterns were analyzed with the X'Pert HighScore program with reference patterns from the International Centre for Diffraction Data's (ICDD's) Powder Diffraction File (PDF)-2 and PDF-4 databases. The X'Pert software was also used to perform Rietveld refinement of each pattern, which allowed quantification of crystalline phases in each sample to an accuracy of 1–3 wt % (the accuracy increases with phase abundance).

### 2.3. Pyrolysis

The thermal decomposition of each sample was investigated by loading 10 mg of each powder into quartz pyrolysis tubes plugged by quartz wool. The tubes were placed in the platinum coil of a CDS 2000 pyroprobe and into a pyrolysis chamber purged with helium, with a flow rate of 14 mL min^−1^. The coil was heated at the maximum rate of 20°C ms^−1^ to 650°C, where it was held for 15 s. The pyrolysis interface was held at 150°C and coupled to a GC-MS for direct injection.

### 2.4. Gas chromatography–mass spectrometry

Gas chromatography–mass spectrometry analyses were conducted with an Agilent Technologies 6890 gas chromatograph coupled to a 5973 mass spectrometer. A solvent delay of 2.5 min was added to the GC-MS method file to prevent excessive amounts of sulfur dioxide from damaging the mass spectrometer. The gas chromatograph injector was held at 270°C and operated in split mode (10:1) with a column flow rate of 1.1 mL min^−1^. Separation was performed on a J&W DB-5MS UI column (30 m × 0.25 mm × 0.25 μm). The gas chromatograph oven was held for 2 min at 40°C and then ramped to 310°C, where it was held for 10 min. Mass spectra were acquired in the scan range 45–550 amu.

### 2.5. Comparison to SAM instrument parameters

The experiments described in this manuscript make use of flash pyrolysis in combination with GC-MS, while the SAM instrument currently operating on Mars uses ramped pyrolysis with EGA and/or GC-MS (Mahaffy *et al.,* 2012; Glavin *et al.,* 2013). SAM has a pyrolysis oven ramp rate of 35°C min^−1^ with a helium flow of approximately 0.8 mL min^−1^ and for EGA a split flow to the mass spectrometer of around 800:1 (Glavin *et al.,* 2013). In contrast to the direct injection used in our methods, SAM makes use of a hydrocarbon trap for GC-MS analysis (Glavin *et al.,* 2013). The SAM gas chromatograph helium flow rate is approximately 0.4 mL min^−1^ with a split of approximately 250:1 (Glavin *et al.,* 2013). The scan range of the SAM quadrupole mass spectrometer is 2–535 amu (Glavin *et al.,* 2013).

## 3. Results

### 3.1. The mineralogy of acidic stream sediments

**FIG. 2.**
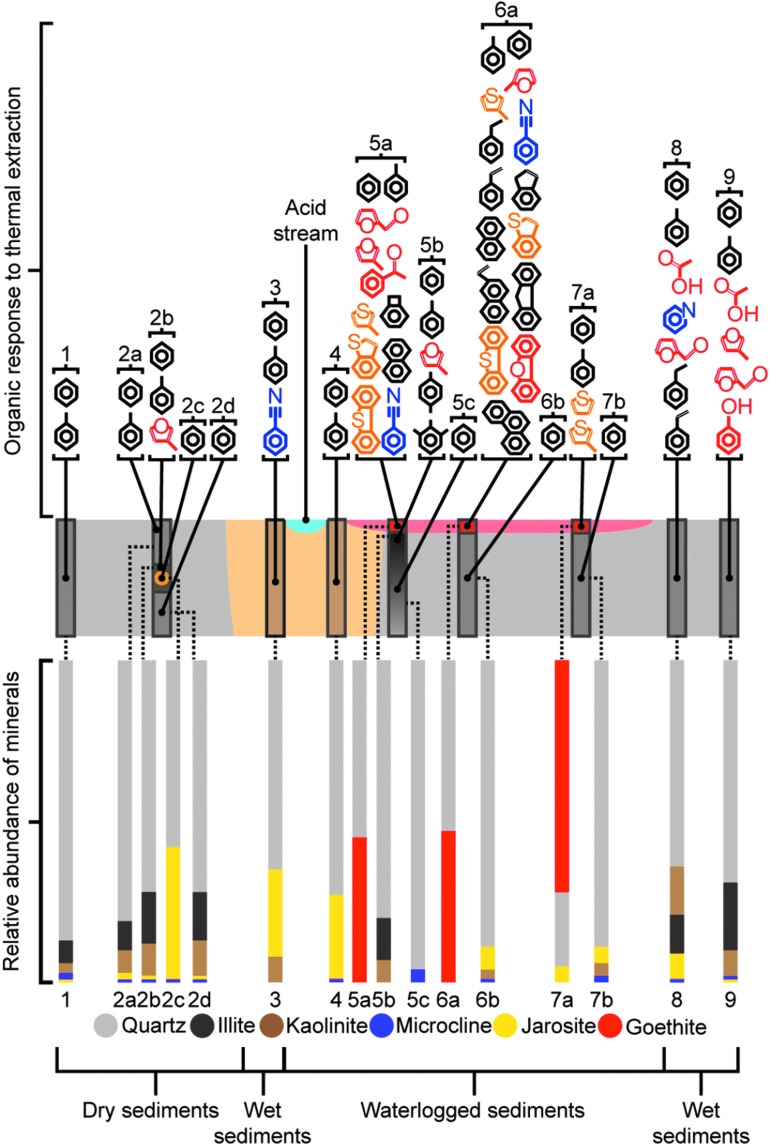
The mineralogy and organic chemistry of the acidic stream samples. The relative abundance of minerals deduced by XRD for each core and sublayer (Table 1) are shown below an idealized cross section of the sulfate ecosystem. Above the cross section, the organic compounds detected during pyrolysis of materials from each core and sublayer are presented (Table 2). Oxygen-containing compounds are highlighted in red, nitrogen-containing in purple, and sulfur-containing in orange.

The acidic stream had a pH of 3.5. The dominant lithology across the study site was a quartz sand with 10–20 wt % clay minerals and minor potassium jarosite and microcline (Fig. 2; Table 1). Core 2 bisected a large fragment of wood that had been buried in the sand. Within the wood, a 2 cm diameter nodule of jarosite was found. Cores 3 and 4 were sampled on each side of the stream and showed high concentrations of jarosite (27 wt % and 26 wt %, respectively). Cores 5–7 were capped with a thin crust rich in goethite. Core 5 did not contain any jarosite. There was a smell of hydrogen sulfide upon extracting Core 5, suggesting conditions became reducing at shallow depths. Core 6 did not host any jarosite in the goethite cap, but jarosite was present at 7 wt % in the quartz sand beneath. Core 7 was the only core to contain a layer where quartz was not the dominant phase, as goethite was present in the cap at 72 wt %, while quartz was present at 23 wt % and jarosite at 5 wt %. Cores 8 and 9 had similar bulk mineral assemblages but with Core 8 hosting more jarosite at 8 wt % compared to 1 wt %.

**Table 1.** Mineral Composition of Each Core and Sublayer (wt %)

	*Core/Sublayer composition (wt %)*															
*Mineral*	*1*	*2a*	*2b*	*2c*	*2d*	*3*	*4*	*5a*	*5b*	*5c*	*6a*	*6b*	*7a*	*7b*	*8*	*9*
Quartz	87	81	72	58	72	65	73	55	80	96	53	89	23	89	64	69
Illite	7	9	16	-	15	-	-	-	13	-	-	-	-	-	12	21
Kaolinite	3	7	10	-	11	8	-	-	7	-	-	3	-	4	15	8
Microcline	2	1	1	1	1	-	1	-	-	4	-	1	-	2	1	1
Jarosite	1	2	1	41	1	27	26	-	-	-	-	7	5	5	8	1
Goethite	-	-	-	-	-	-	-	45	-	-	47	-	72	-	-	-

When jarosite was present at 5 wt % or greater, the unit cell parameters could be used to estimate the relative abundance of K-Na-H_3_O in jarosite by using the method of Basciano and Peterson (2008). All of the jarosite sampled, other than the jarosite nodule in Layer 2c, was enriched in potassium with minor sodium and hydronium. The 2c nodule had similar amounts of hydronium but with major sodium and potassium. During sampling of the acidic stream sediments, crusts of jarosite were observed on surface wood fragments in close proximity to Core 2. These were sampled, and XRD revealed that the crusts were composed of natrojarosite, gypsum, and metasideronatrite, suggesting a formation related to sea spray (Moreton *et al.,* 1995; Garvie, 1999). The nodule observed in 2c was likely a crust originally present on wood at the surface that was then buried and partially enriched in potassium jarosite by fluids from the acidic stream (Brophy and Sheridan, 1965). These findings illustrate how XRD can distinguish different fluid origins for closely associated jarosite units when the iron sulfate is present at sufficient abundance. The XRD patterns showed no indications for amorphous phases or species such as schwertmannite or ferrihydrite, which can be metastable phases in the jarosite-goethite system (Stoffregen *et al.,* 2000).

### 3.2. Pyrolysis studies of biological materials

A number of different types of biological materials, and therefore sources of organic matter, were observed across the study site. Grasses were present near Cores 1 and 2, and when pyrolyzed they produced species such as phenols, styrene, fatty acids, and larger compounds such as cyclotetracosane and stigmastandiene. Fragments of wood were present on the surface and gave a diverse response for phenols and fatty acids, in addition to styrene and a glucopyranose sugar. Pyrolysis of the microbial mat produced compounds such as benzonitrile, glucopyranose sugars, fatty acids, maltol, naphthalene, phenols, isoquinoline, and benzothiophenes. Benzene, toluene, furan, and furfural were ubiquitous pyrolysis products for all the biological materials.

### 3.3. Pyrolysis studies of mineral layers

Cores 1–3, to the west of the acidic stream, produced very weak peaks for benzene and toluene during pyrolysis (Fig. 2; Table 2). Layer 2b, which was the layer of wood bisected by the core, also produced 3-methyl-furan when heated. When compared to Core 1 and Core 2, Core 3 gave the strongest response for benzene and also yielded benzonitrile. The sands collected in close proximity to the grass-covered slopes to the east of the stream produced benzene and toluene but also acetic acid, furan, styrene, and phenol (Cores 8 and 9). The goethite-rich crust present in the waterlogged soils to the east of the stream gave a significantly greater response for organic compounds during heating (Cores 5 to 7). The pyrolysis products included dibenzofuran, fluorene, indene, naphthalene, phenanthrene, thiophenes, and benzothiophenes (Table 2). The pyrolysis data from the goethite crust were compared to the microbial mat data, as the two were stratigraphically in contact. The goethite crust organic data are dominated by aromatics with few functional groups and compounds such as benzothiophenes and benzofuran. In comparison, the microbial mat produced benzothiophenes and benzofuran but also aromatics with carboxyl, carbonyl, and hydroxyl functional groups as well as fatty acids, tridecene, and pentadecanenitrile.

**Table 2.** Organic Compounds Detected in the Pyrolysis Data for Each Core and Sublayer

	*Core/Sublayer*															
*Organic species*	*1*	*2a*	*2b*	*2c*	*2d*	*3*	*4*	*5a*	*5b*	*5c*	*6a*	*6b*	*7a*	*7b*	*8*	*9*
Methylfuran			**✓**					**✓**	**✓**		**✓**					**✓**
Benzene	**✓**	**✓**	**✓**	**✓**	**✓**	**✓**	**✓**	**✓**	**✓**	**✓**	**✓**	**✓**	**✓**	**✓**	**✓**	**✓**
Acetic acid															**✓**	**✓**
Toluene	**✓**	**✓**	**✓**			**✓**	**✓**	**✓**	**✓**		**✓**		**✓**		**✓**	**✓**
Pyridine															**✓**	
Furfural								**✓**							**✓**	**✓**
Ethylbenzene											**✓**				**✓**	
Xylene									**✓**							
Styrene											**✓**				**✓**	
Trimethylbenzene									**✓**							
Bicyclooctatriene								**✓**								
Benzaldehyde								**✓**								
Benzonitrile						**✓**		**✓**			**✓**					
Phenol																**✓**
Indene											**✓**					
Napthalene								**✓**			**✓**					
Ethenylnapthalene											**✓**					
Dibenzofuran											**✓**					
Fluorene											**✓**					
Phenanthrene											**✓**					
Thiophene													**✓**			
Methylthiophene								**✓**			**✓**		**✓**			
Benzothiophene								**✓**			**✓**					
Dibenzothiophene								**✓**			**✓**					

## 4. Discussion

**FIG. 3.**
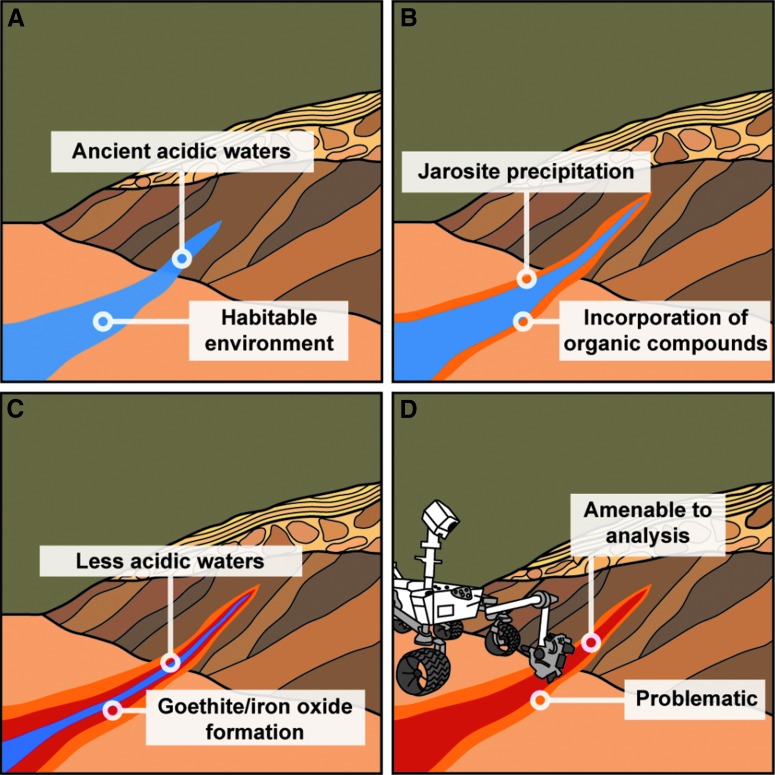
(**A**) Acidic sulfate-rich waters on ancient Mars were potentially habitable environments. (**B**) Jarosite precipitating in these waters may have incorporated organic matter. (**C**) At pH values greater than 3 and high water-to-rock ratios, jarosite has a close association with goethite. Hematite can form by dehydration of goethite or directly from jarosite at low water activities or high phosphate concentrations. (**D**) Unlike jarosite, goethite and hematite do not release substantial amounts of oxygen during thermal experiments. Goethite and iron oxide units formed in close association with jarosite may store an organic record on Mars that is accessible to pyrolysis experiments.

The mineralogical data obtained from the acidic stream samples illustrate the close association between jarosite and goethite. Jarosite will only form without associated goethite when the water-to-rock ratio or pH are low (Zolotov and Shock, 2005). At the acidic stream sample site, dry soils to the west of the stream and the sediments immediately surrounding the acidic waters hosted jarosite but not goethite. In the waterlogged soils to the east of the stream, jarosite was capped by a layer rich in goethite. Though a pristine jarosite outcrop on Mars is extremely important for providing environmental context, it may be of less astrobiological significance than an outcrop where crystalline jarosite and goethite are co-associated. Such an outcrop would suggest a longer-duration aqueous event and likely less acidic conditions as well as being more amenable to pyrolysis studies seeking organic matter (Fig. 3) (Zolotov and Shock, 2005; Lewis *et al.,* 2015).

There has been substantial interest in jarosite on Mars and its ability to preserve organic matter (Aubrey *et al.,* 2006; Skelley *et al.,* 2006; Edwards *et al.,* 2007; Kotler *et al.,* 2008; Marlow *et al.,* 2011; Summons *et al.,* 2011). The data from the acidic stream highlight that goethite should also be strongly considered a target for pyrolysis experiments seeking organic matter on Mars. In terrestrial environments, reactive iron phases such as goethite can bind to organic matter and aid its preservation (Lalonde *et al.,* 2012). Spectroscopy of sediments from Río Tinto, Spain, revealed the preservation of biomolecules in goethite and hematite units formed diagenetically from jarosite (Preston *et al.,* 2011). Incipient fossils, formed during sediment deposition, are also well preserved in Río Tinto iron deposits (Fernández-Remolar and Knoll, 2008). Microbial filaments mineralized by goethite have been observed in the Brick Flat Gossan in Iron Mountain, California (Williams *et al.,* 2015). Ferrihydrite layers located beneath microbial mats at the Chocolate Pots hot springs in Yellowstone National Park were found to host lipid biosignatures (Parenteau *et al.,* 2014). Ferrihydrite is metastable and will recrystallize to goethite or hematite (Parenteau *et al.,* 2014).

The microbial mat overlying the goethite crust was sampled in Cores 5–7 and produced a multitude of organic compounds during pyrolysis. However, for the goethite layers underneath the microbial mat, Core 7 produced only a small range of organic compounds relative to Core 5 and Core 6. Core 7 was the only goethite crust sample where jarosite and goethite were present in the same layer. Sulfur dioxide from jarosite decomposition was observed during pyrolysis despite the solvent delay. Oxygen released by sulfate decomposition will have oxidized organic compounds during pyrolysis (Lewis *et al.,* 2015). The fact that thiophenes were the only organic compounds, other than benzene and toluene, to be detected in Core 7 (Fig. 2) is significant for Mars. Thiophenes are more resistant to oxidation than most other organic species and may therefore survive the oxidizing influence of perchlorates and sulfates during pyrolysis (Otsuki *et al.,* 2000). Thiophenes in pyrolysis data may be indicative of a richer organic inventory that could be unlocked if the influence of oxidizing species is mitigated. However, thiophenes can also form by gas phase reactions in sample ovens (Eigenbrode *et al.,* 2015). It should be noted that the flash pyrolysis methods used in the experiments described in this manuscript are the worst-case scenario for interactions between phases during heating. The negative impact of oxidizing species may be reduced somewhat by ramped heating experiments, such as in an evolved gas analysis when the volatile phases in a sample are not all thermally decomposing simultaneously. For example, the SAM instrument on board MSL uses a ramped heating rate of 35°C min^−1^ (Glavin *et al.,* 2013).

Attempting to thermally decompose oxidizing salts and organic matter at different pyrolysis steps is one potential improvement for analyzing outcrops rich in sulfur and iron on Mars. Another is careful selection of optimal sampling sites. Sampling jarosite outcrops for pyrolysis experiments could provide environmental context and data for potassium-argon dating experiments but would likely be extremely challenging for interpretations about organic matter (Stoffregen *et al.,* 2000; Lewis *et al.,* 2015). The data from the acidic stream indicate that targeting a goethite unit associated with jarosite, but with the goethite unit itself being jarosite-free, may lead to greater success. Ideally both the envisioned jarosite-rich and goethite-rich layers would be sampled individually, but if mission time and resources are limited and organic detection is the mission focus, sampling the goethite layer should take preference.

The data obtained from the acidic stream, in combination with the fact that goethite is known to bind with organic matter, indicate that a goethite unit associated with jarosite on Mars may represent one of the best opportunities to study organic compounds within iron- and sulfur-rich sediments by pyrolysis (Lalonde *et al.,* 2012). However, hematite appears to be far more prevalent on Mars than goethite (Klingelhöfer *et al.,* 2005; Squyres and Knoll, 2005; Zolotov and Shock, 2005; Squyres *et al.,* 2006; Fraeman *et al.,* 2013; Vaniman *et al.,* 2014; Rampe *et al.,* 2016). Some martian hematite units may have formed from the dehydration of goethite or directly from jarosite (Zolotov and Shock, 2005; Barrón *et al.,* 2006). If the water-to-rock ratio was great enough, then jarosite may have been completely converted to goethite or hematite (Zolotov and Shock, 2005). Observations by the Compact Reconnaissance Imaging Spectrometer for Mars (CRISM) instrument on Mars Reconnaissance Orbiter indicate that there are locations within MSL's study site of Gale Crater where hematite is stratigraphically associated with hydrated sulfate layers (Milliken *et al.,* 2010; Fraeman *et al.,* 2013). MSL is well equipped to analyze the geological history of hematite outcrops in Gale Crater (Fraeman *et al.,* 2013). If features such as Vera Rubin Ridge are revealed to have formed from goethite and jarosite precursors, then the data from the acidic stream support sampling of these units and analysis by MSL's SAM instrument. The pyrolysis data produced will develop our understanding of organic preservation in the iron-rich and sulfur-rich sediments that formed in the Late Noachian and Hesperian on Mars.

## 5. Conclusions

The potential for jarosite on Mars to preserve organic matter has received a great deal of recent attention. However, jarosite is problematic for the pyrolysis experiments used by landers and rovers to search for organic compounds, as it releases oxygen during thermal decomposition. Jarosite typically has a close association with goethite and/or hematite, except in low pH environments with low water/rock ratios. The mineralogy of the acidic stream (pH 3.5) studied in this work was dominated by a quartz sand with 10–20 wt % clay minerals and minor potassium jarosite and microcline. A goethite crust covered part of the study site. Samples in which goethite and quartz were the only phases detectable by XRD yielded species such as thiophenes, indene, fluorene, dibenzofuran, naphthalene, and phenanthrene during pyrolysis. Samples containing jarosite typically only produced compounds such as benzene and toluene. When both goethite and jarosite were present in a sample, the organic response was significantly weaker than for the goethite-rich samples that did not contain jarosite. Therefore, when sampling sediments on Mars for organic matter, units that are goethite-rich and associated with jarosite, but not containing jarosite themselves, should be pyrolyzed. Effective sampling of iron-rich and sulfur-rich sediments is of great importance and extremely timely, as exploration targets for the Mars Science Laboratory rover within Gale Crater include Vera Rubin Ridge and hydrated sulfates.

## Abbreviations Used

EGA: evolved gas analysis
GC-MS: gas chromatograph–mass spectrometer, gas chromatography–mass spectrometry
MERs: Mars Exploration Rovers
MSL: Mars Science Laboratory
SAM: Sample Analysis at Mars
XRD: X-ray diffraction
